# Transcriptome variation along bud development in grapevine (*Vitis vinifera* L.)

**DOI:** 10.1186/1471-2229-12-181

**Published:** 2012-10-05

**Authors:** José Díaz-Riquelme, Jérôme Grimplet, José M Martínez-Zapater, María J Carmona

**Affiliations:** 1 Instituto de Ciencias de la Vid y del Vino (CSIC, Universidad de La Rioja, Gobierno de La Rioja), CCT, C/ Madre de Dios 51, Logroño, 26006, Spain; 2 Departamento de Biotecnología, Escuela Técnica Superior Ingenieros Agrónomos, Universidad Politécnica de Madrid, Avenida Complutense s/n, Madrid, 28040, Spain

**Keywords:** Grapevine, Bud development, Dormancy, Flowering, Flower development, Transcriptomics

## Abstract

**Background:**

Vegetative buds provide plants in temperate environments the possibility for growth and reproduction when environmental conditions are favorable. In grapevine, crucial developmental events take place within buds during two growing seasons in consecutive years. The first season, the shoot apical meristem within the bud differentiates all the basic elements of the shoot including flowering transition in lateral primordia and development of inflorescence primordia. These events practically end with bud dormancy. The second season, buds resume shoot growth associated to flower formation and development. Gene expression has been previously monitored at specific stages of bud development but has never been followed along the two growing seasons.

**Results:**

Gene expression changes were analyzed along the bud annual cycle at eight different time points. Principal Components Analysis (PCA) revealed that the main factors explaining the global gene expression differences were the processes of bud dormancy and active growth as well as stress responses. Accordingly, non dormant buds showed an enrichment in functional categories typical of actively proliferating and growing cells together with the over abundance of transcripts belonging to stress response pathways. Differential expression analyses performed between consecutive time points indicated that major transcriptional changes were associated to para/endodormancy, endo/ecodormancy and ecodormancy/bud break transitions. Transcripts encoding key regulators of reproductive development were grouped in three major expression clusters corresponding to: (i) transcripts associated to flowering induction, (ii) transcripts associated to flower meristem specification and initiation and (iii) transcripts putatively involved in dormancy*.* Within this cluster, a MADS-box gene (*VvFLC2*) and other transcripts with similar expression patterns could participate in dormancy regulation.

**Conclusions:**

This work provides a global view of major transcriptional changes taking place along bud development in grapevine, highlighting those molecular and biological functions involved in the main events of bud development. As reported in other woody species, the results suggest that genes regulating flowering could also be involved in dormancy regulatory pathways in grapevine.

## Background

Woody perennial plant species have specific morphological and physiological constraints when compared with annual plants, leading to different reproductive and somatic developmental strategies. Polycarpic woody plants develop terminal or axillary buds with embryonic shoots from which complete branches can develop after specific signals [1]. To elude unfavorable environmental conditions, these buds become dormant providing the possibility to resume growth under viable conditions. Three dormancy states have been distinguished in buds: (i) Paradormancy, induced by distal organs of the plant; (ii) Endodormancy, due to signals internal to the bud itself and (iii) ecodormancy, when bud growth is prevented by environmental factors such as low temperatures [2,3].

Bud para/endodormancy transition (also known as endormancy onset) is generally triggered by environmental factors. Among them, day length and temperature are stable annual cues regulating this process in many plant species in temperate regions. Endo/ecodormancy transition (or endodormancy release) requires the completion of a chilling period that leaves the bud in an ecodormant stage, susceptible to initiate bud break upon a period of favorable temperatures [4-7]. Regulation of endodormancy onset and release involves plant hormones such as auxins, ethylene, abscisic acid and gibberellins which could also interact with sugars signaling [4,6]. Epigenetic regulation throughout chromatin modification has also been proposed to be involved in dormancy regulatory processes based on differential expression of several chromatin modifying proteins [5].

In woody species that set dormant terminal buds, cessation of vegetative growth precedes the onset of dormancy and the terminal bud differentiates directly from the shoot apical meristem (SAM). Thus, bud formation is concomitant with dormancy onset [6-8]. In other woody species, including grapevine, axillary buds become dormant when perceiving the environmental signals triggering the dormancy onset, whereas SAM ceases growth when environmental conditions become unfavorable [9,10].

Little is known about the molecular events underlying bud dormancy in woody species. Photoperiodic regulation of dormancy could involve *Phytochromes* (*PHY*) and the circadian clock since over-expression of *PHYA* prevents short day (SD)-photoperiod endodormancy induction in *Populus*[11,12]. In addition, it has been proposed that signaling pathways regulating dormancy onset and release may share genetic components with flowering regulation, for example members of the *Flowering Locus T* (*FT*)*/ Terminal Flower1* (*TFL1*) and the MADS-box gene families [5]. Both *FT* and *TFL1* homologs are repressed in *Populus* and leafy spurge by environmental factors inducing dormancy [5,11,12]. Moreover, over-expression of *PHYA* in transgenic *Populus* prevents *FT* and *TFL1* homologs repression and the onset of dormancy [12]. Finally, both genes are up regulated by the chilling temperatures causing endodormancy release [13].

Some members of the MADS-box transcription factors family, such as *Flowering Locus C* (*FLC*) and *Short Vegetative Phase* (*SVP*), involved in the temperature regulation of flowering response in Arabidopsis [14,15], could participate in the regulation of bud dormancy in woody perennial species. *FLC* homologs are up regulated during endodormancy and their expression decreases after dormancy release both in *Poncirus* and leafy spurge [16,17]. Similarly, *SVP* homologs, known as *Dormancy Associated MADS*-box (*DAM*) genes, have also been involved in growth cessation and terminal bud formation in woody perennial plants such as peach, raspberry, kiwi, apricot, leafy spurge, *Poncirus* or *Populus*[18-24]. The peach *evergrowing* (*evg*) mutant, has been shown to carry a deletion of six *SVP*-like genes (*PpDAM1–6*) resulting in a complete lack of dormancy of the terminal shoot meristems [18]. Considering these evidences, it has been proposed that *FLC* and *SVP*-like genes could act by repressing *FT* expression, as they do during flowering in Arabidopsis [15]. This would provoke growth cessation and/or endodormancy [5,17].

In grapevine, winter dormant buds develop from basal axillary buds of lateral shoots. These shoots initiate from prompt buds in the same growing season. Dormant buds are complex bract-protected organs constituted by a primary bud and one or two additional secondary buds [9,10,25]. Growth and development of these buds are initially prevented by paradormancy signals from the apex. However, these latent buds maintain active processes of cell division and differentiation until perceiving (at the end of the summer) the SD photoperiods and temperatures drop that would trigger the onset of dormancy in grapevine [2,25]. Endodormancy has been shown to end when buds have already experienced enough chilling [25,26]. However, buds remain ecodormant until temperature is permissive. When ecodormancy is released, buds swell and the shoot apical meristems (SAMs) in the primary bud follows a program of organ differentiation and growth that gives rise to the new season shoots [10,27]. In Northern hemisphere, the para/endodormancy transition starts in August and the dormant state is released during November [28]. Flowering induction in grapevine takes place in latent buds during the first growing season, whereas flower meristems and flower organs will develop during the second growing season in the consecutive year after bud break [10,29,30]. Processes of inflorescences and flowers initiation and development have been widely reviewed in grapevine [31-33].

Recent works have studied transcriptional characterization of bud responses to chilling [34], photoperiod [35] and dormancy breaking treatments [36,37]. Their results have led to the identification of candidate genes with a dual role in flowering and dormancy in *Vitis riparia*[34,35] and to propose of a role for oxidative stress as part of the dormancy releasing mechanisms [36-38].

In this study we have followed the grapevine bud transcriptome along the complete annual cycle which includes two growing seasons. Bud transcriptome analyses identified three major phases of transcriptome change, mainly associated to dormancy. Furthermore, the expression of members of the MIKC-type MADS-box gene family [39], the *SPL* (*Squamosa Promoter Binding Protein-Like)* family, the *FT-TFL1* family [31] and the *VFL* gene (the grapevine *Floricaula/Leafy* ortholog) [40], was also analyzed during bud development.

## Results and discussion

### Bud transcriptome variation along the annual cycle

**Figure 1:**
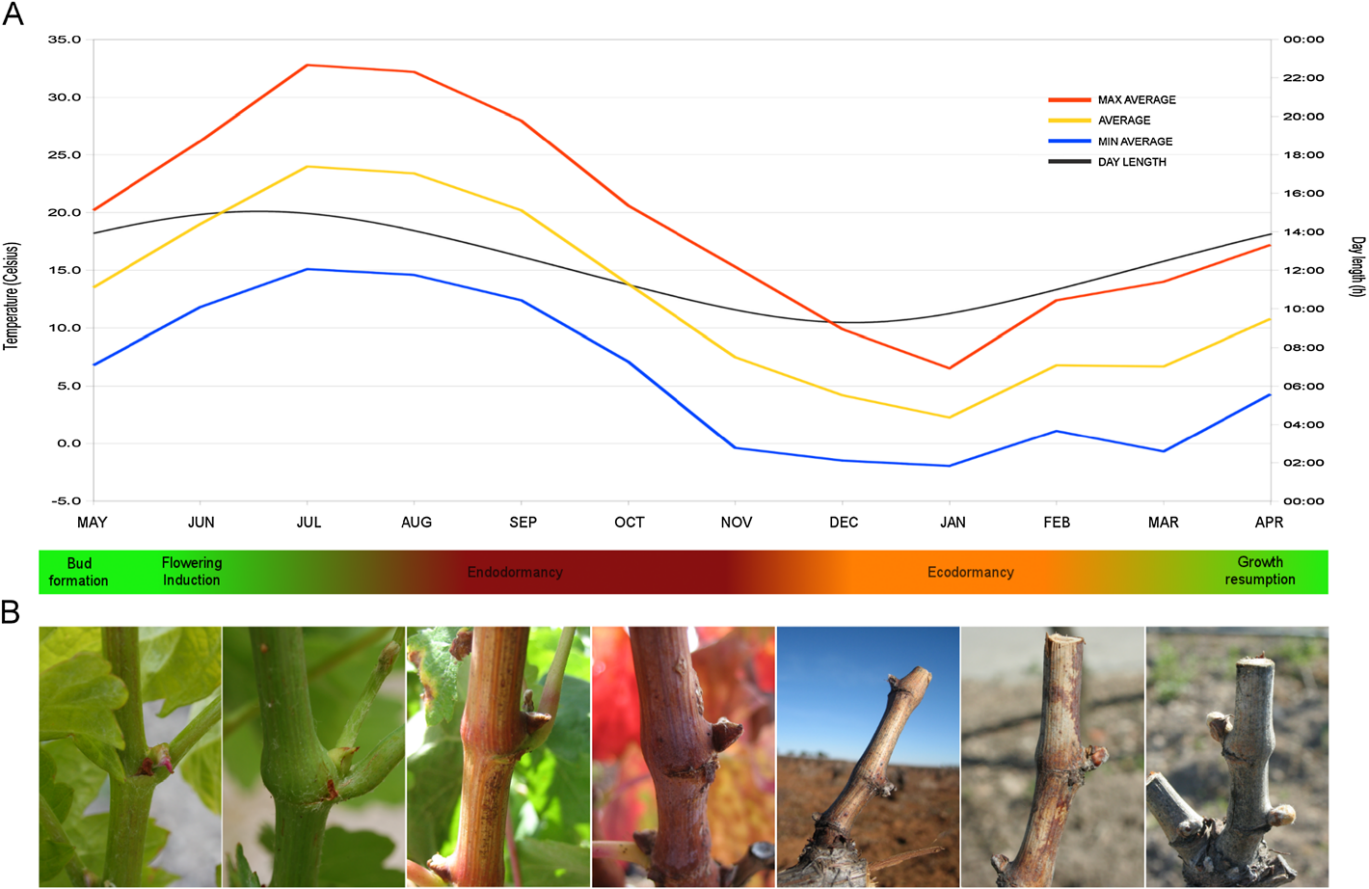
**Bud developmental evolution along the year.A**) Environmental change in temperature and day length. **B**) Pictures depicting grapevine axillary buds along the year, corresponding to May, June and July, dormant buds from September to March, and during bud break in April. Temperatures are shown as monthly minimum average (blue line), monthly maximum average (orange line) and monthly average (yellow line). Day length (grey line) is shown as light hours per day. Assignments of bud stages were based on previous data on cv. Tempranillo [40,41].

Grapevine bud development is modulated by environmental factors such as temperature and day length [25]. In our experimental conditions, Tempranillo cv. latent buds are formed during the first growing season in the young sprouting stems between April and May (APR-MAY) and experience active developmental processes involved in the set up of the vegetative and reproductive growth until the end of the summer of the following year (Figure 1) [32]. Flowering induction takes place within latent buds around the middle of June (JUN) and inflorescence primordia differentiate from lateral meristems developed by the shoot apex. Inflorescence meristems proliferate to generate inflorescence branch meristems in complex inflorescence primordia along July (JUL) and August (AUG) [40]. In our growing conditions, Tempranillo buds are endodormant in the second half of September (SEP) and endodormancy is released by the end of November (NOV), although buds remain in an ecodormant stage from DEC to MAR [41]. Once winter is over, ecodormancy is released and inflorescence branch meristems proliferate to produce flower meristems in April (APR) swelling buds that initiate the second growing season [40].

**Figure 2:**
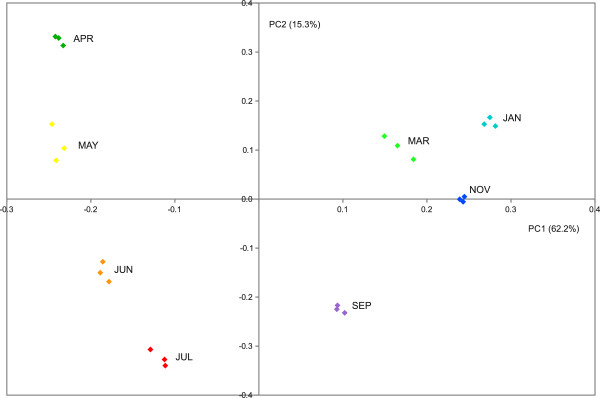
**Bi-dimensional loading score plot of sample replicates resulting from PCA analysis.** X axis represents PC1 that explains 62.2% and Y axis represents PC2 that explains 15.3% of the total variability for gene expression. Samples belonging to the same time-point are represented by the same color.

High throughput transcriptional analysis was performed along bud development on bud samples collected at 8 different time points during the annual cycle (see Methods). Principal Components Analysis (PCA) was performed on the whole expression dataset (Additional file 1) to verify correlation among different biological replicates and to identify main sources of gene expression variation. The results of the PCA plot showed consistency across biological replicates, as shown in Figure 2. The first two principal components (PC1 and PC2) explained 77.5 percent of the total variability in gene expression (62.2 percent and 15.3 percent respectively). PC1 seems to represent the time course evolution of bud developmental stages and appears to be reset to the original status with bud swelling, since APR bud samples of the second season were neighboring to MAY bud samples of the first season (in the same quadrant), revealing a high transcriptome similarity between those two stages. PC2 highlights major transcriptome differences between JUN, JUL and SEP samples and the remaining time points.

**Figure 3:**
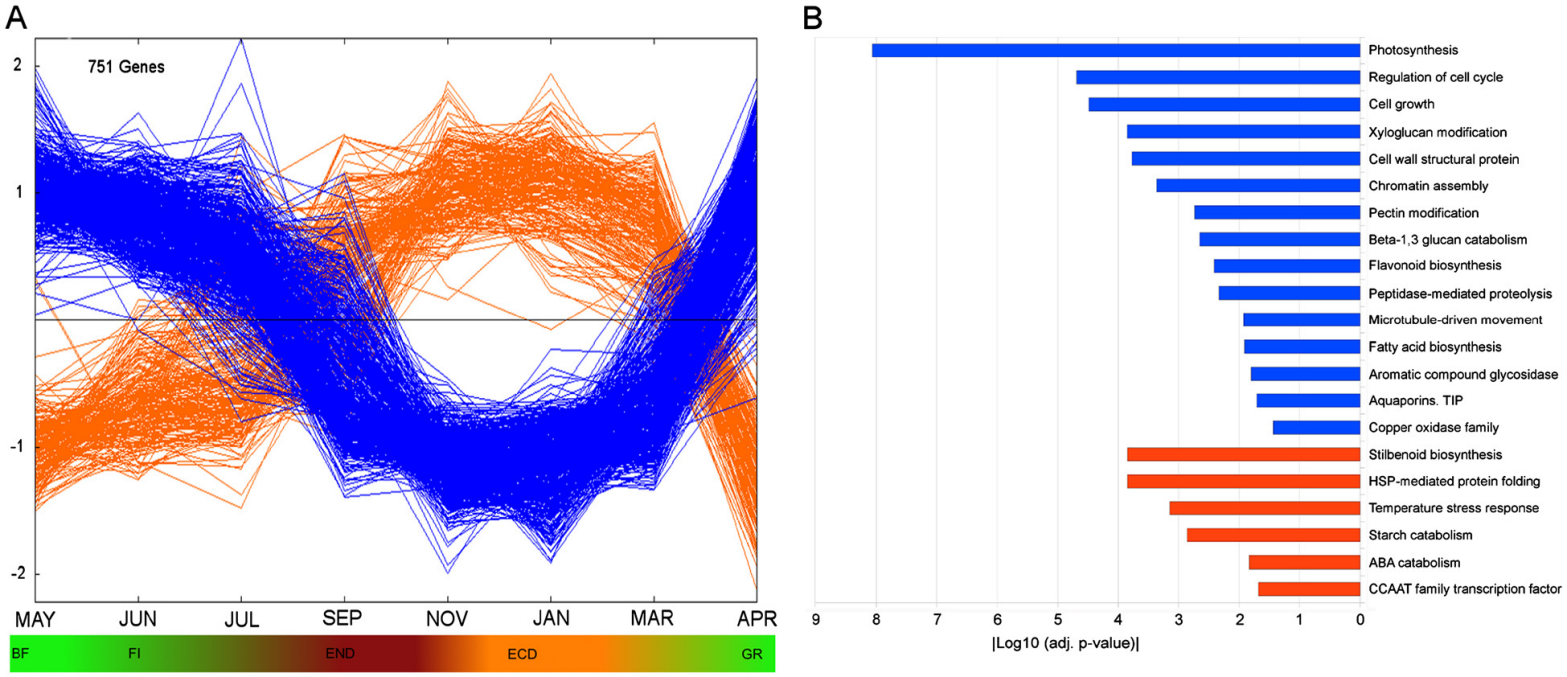
**Top-scored transcripts for Principal Component 1.A**) Expression profiles of the transcripts with positive component score values (orange lines) and negative component score values (blue lines). Each single line represents the average of mean-centered expression values for an individual transcript. **B**) Functional categories over-represented in each cluster. Color code is the same as in A. Absolute values of the log_10_ transformed *P*-values were used for the bar diagram representing statistical signification, only categories with *P*-values < 0.05 were shown. BF, bud formation; FI, flowering induction; END, endodormancy; ECD, ecodormancy; GR, growth resumption.

To investigate the biological basis of the principal components, transcripts with the highest contribution to each component in the analysis were identified according to the absolute value of their component score (CS) for PC1 and PC2 (Additional files 2 and 3). Figure 3A shows the expression profiles of transcripts mostly contributing to PC1. Transcripts with negative CS values (547 transcripts, blue color) are up-regulated in non-dormant buds and show declining expression during endodormancy and the lowest expression during ecodormancy. Transcripts with positive CS values (204 transcripts, orange color) follow the opposite trend (Figure 3A). Functional enrichment analyses indicated that the transcripts up-regulated in non-dormant buds were characteristic of actively proliferating and growing cells (Figure 3B). Among them, transcripts related with cell division, cell growth and differentiation (cell-cycle regulation, microtubule-driven movement, chromatin assembly, cell growth, peptidase-mediated proteolysis and cell wall metabolism) and those related with primary and secondary metabolism (photosynthesis, fatty acid biosynthesis, flavonoid biosynthesis and aromatic compounds glycosidation) showed high CS value.

On the other hand, the most significant functional categories contributing to PC1 and enriched in dormant buds (orange color) were those related to stress responses (stilbenoid biosynthesis, "HSP-mediated protein folding", temperature stress response, as well as CCAAT transcription factor family) [42]. These functions could be related to bud responses to dehydration and temperature changes that take place together with dormancy, as previously reported in *Populus*[24]. Furthermore, the observed up-regulation of genes involved in ABA catabolism could be related to the decay of this hormone previous to dormancy release [5]. Finally, an increase in the expression of starch catabolism genes together with a down-regulation of genes encoding photosynthetic proteins, also observed in *Populus*[24], is in agreement with the physiological state of dormant buds.

Regarding PC2, the enriched functional categories contributing to its negative values were those related to stress responses characteristic of JUN, JUL and SEP samples, whereas the functional categories associated with its positive values were those related to cell proliferation that show higher expression in non-dormant buds (Additional file 4). Therefore, PC2 could represent the transcriptional effects of the stress experienced by buds from JUN to SEP with respect to the remaining stages.

In summary, our results suggest that active growth, dormancy and stress responses are major contributors to the gene expression variability observed along the bud annual cycle. Processes characteristic of actively proliferating and growing cells are up-regulated in non-dormant buds and decline during bud dormancy together with the up-regulation of stress response pathways.

### Transcriptome changes in bud developmental transitions

**Figure 4:**
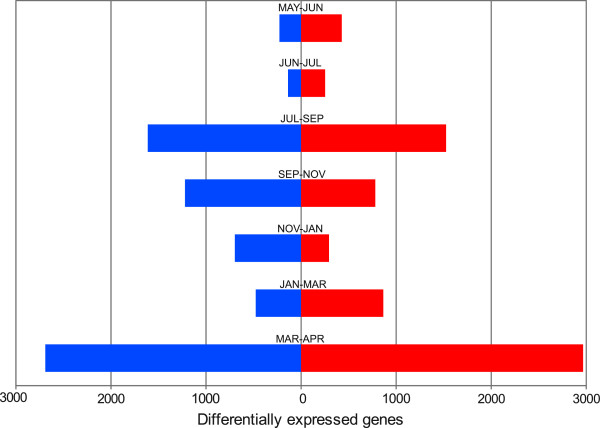
**Differential expressions between consecutive bud time points.** The histogram represents the number of differentially expressed transcripts between each consecutive sample pair, sense of the bars indicates whether transcripts were differentially expressed in the earlier versus later stage (on the left side in blue) or in later stage versus the earlier one (on the right side in red). Numbers correspond to the number of non redundant transcripts in each class.

In order to identify the developmental stages representing major transcriptome changes during the annual cycle of the bud, we performed a pair wise differential expression analysis between consecutive time points. The number of differentially expressed genes varied strikingly among sample pair comparisons (Figure 4). Major changes were observed between JUL and SEP (involving 3139 transcripts), SEP and NOV (involving 2002 transcripts) and MAR and APR (involving 5658 transcripts). Interestingly, these transitions could be associated, respectively, to the proposed timing for para/endodormancy, endo/ecodormancy and ecodormancy/bud break transitions [28,41]. These results supported the conclusions of the PCA experiment, suggesting that the transitions between bud dormancy and active growth explains most of the variation in bud gene expression profiles.

**Figure 5:**
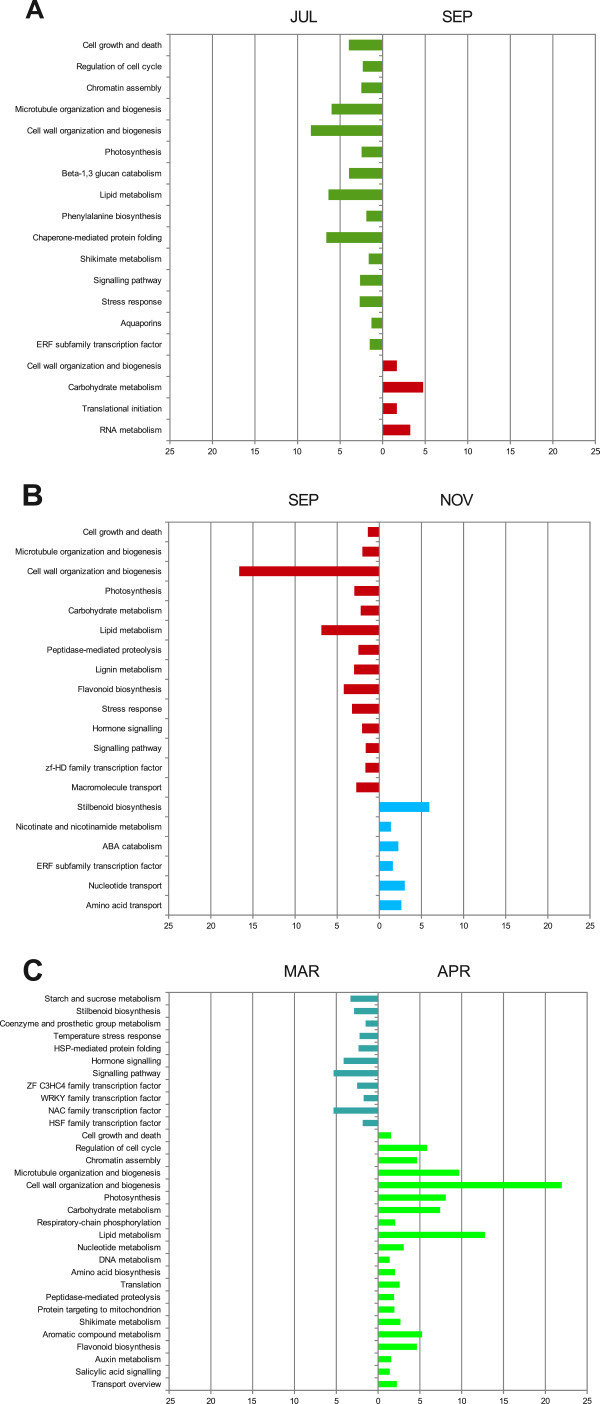
**Functional categories over-represented in the pair wise comparisons.** Bar chart summarizes the significantly enriched functional categories between stages flanking these transitions. The sense of the bars indicate whether transcripts were differentially expressed in the earlier versus later stage (on the left side) or in later stage versus the earlier one (on the right side). Absolute values of the log_10_ transformed *P*-values (of the enrichment analysis) were used for plotting, only categories with *P*-values lower than threshold (0.05) were shown.

In order to identify biological functions involved in the three major bud transcriptional changes we performed studies of functional categories enrichment (Figure 5). The para/endodormancy transition (JUL to SEP) (Figure 5A) was characterized by a major reduction in enriched functional categories that mostly contributed to PC1 in Figure 3. Among them, categories related to cell proliferation (including regulation of cell cycle, chromatin assembly and microtubule organization and biogenesis) and cell growth and death, were down regulated from JUL to SEP. These results are consistent with the shutdown of those processes during bud dormancy that has been described in other systems [24,43]. Cell wall organization and biogenesis, also linked to cellular processes, was significantly reduced with endodormancy. Modification of the cell wall, mainly xyloglucans metabolism and cell wall proteins, was an enriched category in JUL samples but not in SEP. However, cell wall biosynthesis (mainly including cellulose biosynthesis transcripts) appeared as a significant category in SEP. Metabolism of carbohydrates was in some aspects related to cell wall metabolism, thus 1,3-β-glucan catabolism predominated in JUL while in SEP carbohydrate metabolism was mainly represented by starch and sucrose metabolism. These results could be related to the required sealing of plasmodesmata with callose to lower their size exclusion limit, a process dependent on 1,3-β-glucansynthase and 1,3-β-glucanase activities. Additionally, cell wall composition must be modified to reduce water and molecules movement among cells during this stage [44].

Representation of photosynthesis-related functional categories also decreased during para/endodormancy transition, in agreement with results reported in *Populus*[24]. Lipid metabolism (including fatty acid biosynthesis, glycerolipds metabolism and oxylipins) was also under-represented in this transition. This change could be related to the formation of lipid bodies (LBs) which store triacylglicerols and improve freezing tolerance previous to endodormancy, as has been reported in other systems [44]. However, as far as we know, no cytological evidence on the formation of LBs exists in grapevine buds.

Other significant functional categories that were under-represented from JUL to SEP were those related to stress responses (mainly abiotic and temperature), chaperone-mediated protein folding and aquaporins (mainly TIP). In *Populus*, genes responsible for adaptation to dehydration and low temperatures have been shown to be expressed in response to SD even in the absence of those stresses [24]. In leafy spurge, genes responsive to cold stress were also up-regulated in fall and winter [43]. Within the signaling pathways functional category, 129 transcripts were down-regulated between JUL and SEP. Among them, salicylic acid-mediated signaling was significantly enriched, what could be related to stress responses as well as elicitation of phytoallexin biosynthesis [45].

The transcription factor functional category was also significantly enriched among transcripts down-regulated in this comparison. Among them, a significant enrichment was also proven for the ERF subfamily (9 transcripts), within the AP2-like [46] transcription factor family, most of them related to ethylene regulated responses. In *Populus*, the temporal expression of some ERF-like transcripts at the beginning of SD photoperiods has suggested a role for ethylene in the regulation of dormancy [24]. In fact, *Populus*, leafy spurge and potato have a transient peak in ethylene or ethylene perception associated with endodormancy induction [5].

Only a few functional categories were significantly enriched among transcripts showing up-regulation from JUL to SEP. Apart from those concerning cell wall organization and biogenesis and carbohydrate metabolism that were previously described, functional categories related to metabolism of nucleotides and nucleic acids and protein metabolism were also significant. Among them, enrichment of RNA metabolism and translation initiation could reveal the existence of mechanisms relying on stored mRNAs transcripts ready to be translated, as described in dry angiosperm seeds [47].

The endo/ecodormancy transition (SEP to NOV) (Figure 5B) was characterized by a further decline in transcripts participating in cellular processes as well as primary and secondary metabolism functional categories, similar to what was observed in the para/endodormancy transition. Functional categories related to cell wall metabolism and biogenesis (mainly based on biosynthesis of cellulose, catabolism of pectins and modification of pectins and xyloglucans) were still relevant in SEP and further down-regulated in NOV. Moreover, carbohydrate metabolism-related transcripts that were down-regulated in SEP versus NOV corresponded now to oligosaccharides metabolism and glucans catabolism. A parallel situation has been reported in *Vitis riparia* during the chilling period required for endodormancy release [34]. Stress responses were also under-represented from SEP to NOV, especially categories related to oxidative stress. An activation of the oxidative stress response machinery preceding endodormancy release would be in agreement with previous reports showing that oxidative stress affecting mitochondrial function could participate in endodormancy release in grapevine [36,37].

On the other hand, the SEP to NOV transition was marked by an enrichment of ABA catabolism, in agreement with the role of ABA in endodormancy and its decay during endo/ecodormancy transition [5]. ABA levels correlate with bud dormancy in several species and decay throughout the transition to ecodormancy. Both in *Populus* and leafy spurge, ABA content peaked after few weeks of SD and decayed later [24,43]. Moreover, an ABA-related transcript has also been reported to be down-regulated during the chilling period required for endodormancy release in grapevine [34]. Buds of NOV also showed an enrichment of stilbenoid biosynthesis that in grapevine usually responds to biotic and abiotic elicitors [48]. Finally, enrichment of nicotinate and nicotinamide metabolism in NOV as well as nucleotide and amino acid transport might suggest the initiation of certain metabolic activity paralleling the endo/ecodormancy transition. Interestingly, the ERF subfamily of transcription factors that was significantly enriched in JUL versus SEP was also found in NOV versus SEP. Moreover, five of these transcripts were common in JUL and NOV, suggesting common functions before para/endodormancy and after endo/ecodormancy transitions.

The ecodormancy/budbreak transition (MAR to APR) (Figure 5C) was characterized by down-regulation of specific functional categories involved in starch and sugar catabolism or signaling and stress responses mainly related to previous periods. Similar underrepresentations were also observed in transcription factor families known to be involved in stress adaptive responses such as HSF [49], NAC [50] and WRKY [51] families, as has been reported in other species [19,43]. In contrast, bud break in APR was characterized by the presence of most functional categories that were enriched in JUL before bud para/endodormancy transition. Those categories were related to cellular processes required for cell proliferation (chromatin assembly, microtubule organization and biogenesis, cell cycle regulation) and cell growth (cell growth and death, cell wall organization and biogenesis, photosynthesis and primary and secondary metabolism). Up-regulation of cell wall organization and biogenesis and carbohydrate metabolism categories (mono, oligo and polysaccharides and more specifically glucans and 1,3-β-glucan catabolism), could also be related to restoring cell wall properties and cell communication throughout callose hydrolysis at plasmodesmata. Induction of 1,3-β-glucanase after the application of dormancy-release agents has also been reported in grapevine [38]. Secondary metabolism categories included enrichment of flavonoid biosynthesis (mainly anthocyanins) as well as aromatic compound and shikimate metabolism as probable flavonoid precursors. Interestingly, the significantly enriched hormone signaling functional categories were those related to auxin and salicylic acid, that likely have a role in cell proliferation and expansion [52] as well as in the response to biotic stress [45].

### Expression profiles of key regulators of reproductive development

**Figure 6:**
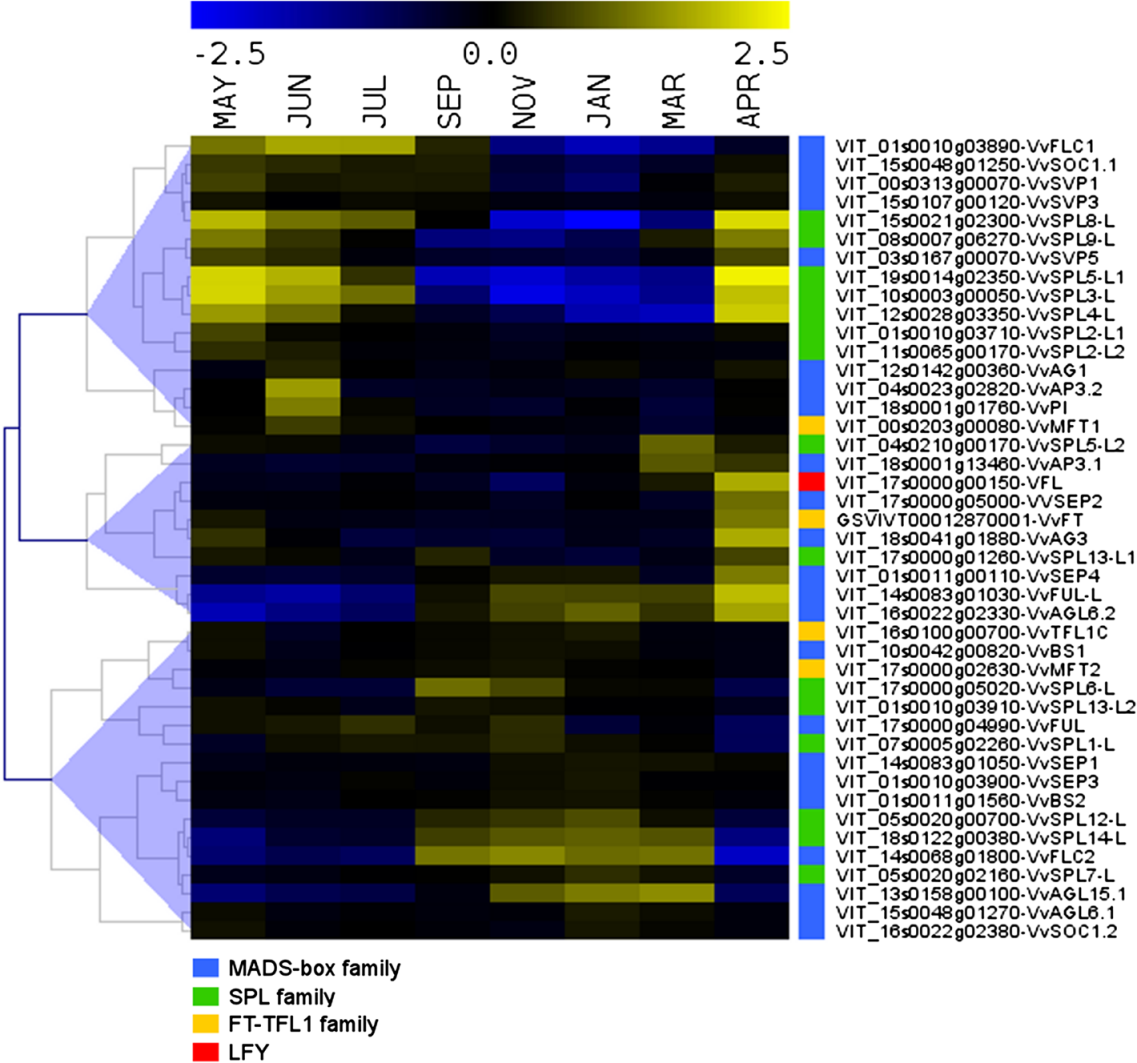
**Hierarchical clustering of expression profiles of key regulators of reproductive development along bud development.** MADS-box gene family (blue boxes), *SPL* gene family (green boxes), *FT*-*TFL1* gene family (orange boxes) and the *VFL* gene (red box). Color scale (on top) represents mean-centered expression values.

Flowering induction in grapevine takes place in latent buds at the beginning of the summer whereas flower meristem differentiation and flower development take place in the second growing season [10,30-33]. To identify putative genes involved in flowering induction and flower development, we examined in detail the expression profiles of reproductive development key genes such as *VFL*, the MIKC-type MADS-box, the *SPL* and the *FT-TFL1* gene families. Hierarchical clustering based on bud expression values of these transcripts along the annual cycle were represented in Figure 6. Consistently with the main bud transcriptional profiles described in the previous section, expression analysis of these transcripts identified three major distinct clusters. The first cluster grouped transcripts up-regulated in non-dormant buds and down-regulated during dormancy. The second cluster contained transcripts with highest expression level during bud break. The third cluster grouped transcripts up-regulated in dormant buds with an opposite expression to those of cluster 1.

Cluster 1 showed transcripts expressed in latent buds when inflorescence primordia are initiated and proliferate. A well characterized gene within this cluster is the MADS-box gene *VvSOC1.1*[39], which expression pattern suggests that it could play a crucial role in flowering induction, as does *SOC1*, its Arabidopsis homolog [53]. Other MADS-box genes in cluster 1 were members of the *FLC* (*VvFLC1*) and *SVP* subfamilies (*VvSVP1*, *VvSVP3* and *VvSVP5*) [39]. *VvFLC1* showed high expression during the first season, decreased during dormancy and increased again during the second growing season (Additional file 5). Expression patterns of the three grapevine *SVP* homologs were distinct than that observed for *SVP* in Arabidopsis [54]. Their expression levels were high at flowering induction, reduced during dormancy and increased again at flower meristem formation. No significant expression changes of *SVP* homologs were observed along grapevine bud dormancy, what would not justify their role in that process, as has been reported in other species [18-24]. Cluster 1 also included *VvMFT1*[31], a member of the *FT-TFL1* family of transcriptional regulators [55,56], with highest expression during the first season. In addition, detection within this cluster of *APETALA3.2*, *PISTILLATA* and *AGAMOUS* homologs suggests that these genes, involved in the specification of flower organs identity, could already be expressed in the inflorescence meristems of first season buds in preparation for the flower organ specification that takes place during the second growing season. Finally, cluster 1 included many homologs of *A. thaliana SPL* genes (*SPL2, SPL3, SPL4, SPL5*, *SPL8* and *SPL9)* what will be discussed later when considering the expression patterns observed within this gene family.

Cluster 2 grouped transcripts showing their highest expression during bud break in the second growing season and likely associated with the events of flower meristems and flower organs differentiation. Consistently with the developmental processes taking place in bud break, cluster 2 contained both *VvFT* and *VFL,* which have homologs in Arabidopsis that are required for flower induction and flower meristem specification. *VvFT* was over-expressed during flowering induction, decreased during bud dormancy and increased again during the second season, which is compatible with the roles proposed for its homologs in *Populus* and other woody plants [7,11,13,43]. Although the putative *TFL1* homolog *(VvTFL1A)* was not present in the Grapegen GeneChip®, our previous analysis of this gene family [31] showed that it is down-regulated during dormancy and up-regulated with dormancy release. This expression pattern parallels what has been reported for *TFL1* homologs in *Populus*[13]. *VvSPL13-L* and *VvAG3* on one side and V*vFUL-L*, *VvAGL6.2* and *VvSEP4* on the other, showed a similar expression pattern and could also participate in the processes of flower meristem and flower organ specification.

Cluster 3 included transcripts showing their highest expression during dormancy. Interestingly, this cluster included two MADS-box genes, *VvFLC2* and *VvAGL15.1,* which Arabidopsis homologs have been involved in flowering repression. In addition, it contains several *SPL* genes (*VvSPL6-L, VvSPL12-L* and *VvSPL14-L*). The remaining genes also showed some expression in non-dormant stages. The two *FLC* homologs found in grapevine (*VvFLC1* and *VvFLC2*) [39] showed an opposite expression pattern in buds (Additional file 5). *VvFLC1* behaved as *PEP1*, the *Arabis alpina FLC* homolog. *PEP1* expression is reduced during the winter and increases when growth is resumed after the cold period [57]*.* Fluctuations in the *FLC* transcript levels in this perennial species would allow some meristems to undergo flowering transition while others maintain the vegetative growth of the plant. A similar role for *VvFLC1* in maintaining vegetative growth of the young meristems that will give rise to the new latent buds could be proposed in grapevine. In contrast, the opposite expression pattern of V*vFLC2*, resembles expression reported for *FLC*-like genes in *Poncirus*[16] and leafy spurge [17], where they could be involved in the regulation of dormancy. Similarly, a role as repressor of flower meristem initiation has been proposed for *AGL15* and *AGL18* in Arabidopsis [58]. Interestingly, their putative grapevine homolog, *VvAGL15.1,* has the same expression pattern as *VvFLC2*. The search of *FLC* gene homologs with opposite expression pattern in other polycarpic plant species could help to elucidate their role in these processes. In addition, two members of the *FT/TFL1* gene family (*VvTFLC1* and *VvMFT2*) [31] were also found in cluster 3, opening the possibility that their function could be related with the control of dormancy in grapevine. In agreement, high expression of a member of the *FT/TFL1* family (*PaFT4)* in Norway spruce (*Picea abies* L.) correlates with growth cessation and bud set [59], in contrast to that observed for the *PtFT1* gene of *Populus*[11]. Indeed, comparative sequence and expression analysis of the *FT/TFL1* family in gymnosperm and angiosperm species lead to speculate that the original function of this gene family could be related to the regulation of growth arrest and/or dormancy [60].

Different members of the *SPL* family of transcription factors were found within the three clusters. This family of transcription factors is known to participate in the regulation of diverse plant developmental processes such as plant phase transition, flower and fruit development and plant architecture [61-64]. Ten of the 16 Arabipdopsis *SPL* genes are post-transcriptionally regulated by miR156, which incorporates endogenous age/development signals into vegetative phase transition and flowering [61,62]. This vegetative phase regulatory mechanism is also conserved in woody perennials [65]. Interestingly, cluster 1 contains transcripts homologous to *SPL3, 4, 5* and *9,* all belonging to the miR156/7-targeted *SPL* subfamily, which act as positive regulators of juvenile-to-adult phase change transition and flowering in Arabidopsis [63,64,66] and are regulated by *SOC1*[67]. Cluster 1 also included two *SPL2* and one *SPL8* homologs. Arabidopsis *SPL2* is also miR156 targeted and seems to be involved in lateral organ development within the reproductive phase [68]. The miR156/7 non-targeted *SPL8* gene is involved in pollen sac development [69] and required for male fertility [70]. Finally, several members of this gene family were found in Cluster 3 displaying an expression pattern more restricted to the dormancy period (*VvSPL1-L, VvSPL6-L, VvSPL7-L, VvSPL12-L, VvSPL14-L and VvSPL13-L2*). Most Arabidopsis counterparts of these genes belong to the miR156/7 non-targeted *SPL* subfamily (*SPL1, 7, 12, 14* and *16*) that comprises the larger proteins in the family [70]. Little is known about the functions of these putative transcriptional regulators with the exception of *SPL14*, which seems to regulate plant architecture and the length of vegetative phase. An Arabidopsis mutant with reduced *SPL14* expression, had elongated petioles, serrated leaf margins and accelerated vegetative phase change [71], suggesting that this gene could play a role as a negative regulator of phase transition and flowering [72], having antagonistic function to other SPL proteins that promote vegetative phase change. Interestingly, both *VvSPL14-L* and *VvSPL12-L* showed an expression pattern very similar to *VvFLC2* and *VvAGL15.1* which could also suggest a role for these *SPL* genes in dormancy maintenance. Little is known about the role of *SPL*-like genes in woody species. Two genes (*SPL*-like *3* and *6*) were detected during dormancy in *Populus*[24] with *SPL6*-like increasing and *SPL3*-like decreasing along dormancy. In addition, an *SPL-2* homolog has also been found in grapevine which seems to be regulated by photoperiod [35]. Further studies will be needed to elucidate the possible role of the *SPL* gene family in bud dormancy.

Additional mechanisms involving transcriptional repressors could be required during the dormancy period to prevent premature flower meristems formation from the inflorescence meristems. The establishment of annual and perennial life has independently arisen several times in flowering plants [73], so it is likely that mechanisms involved in the control of bud dormancy or repression of flower meristem formation have recruited different regulatory genes in different botanical families. Complementary experiments will be needed to assess the biological function of the members of these transcriptional regulators families in such processes.

## Conclusions

Transcriptional analyses along bud development have shown that principal components explaining the observed expression variability are determined by genes involved in active cell growth and proliferation, dormancy regulation and stress responses, indicating that these are the most active events in bud development. Major transcriptional changes were detected between samples collected in July and September (para/endodormancy transition), September and November (endo/ecodormancy transition) and March and April (ecodormancy/bud break transition). The functional categories enriched in these transitions are in agreement with the results of the Principal Component analysis.

Expression profiles of key regulators of reproductive development were assigned to three major transcriptional clusters corresponding to (i) transcripts associated only to flowering induction; (ii) transcripts associated to flowering induction and flower meristem initiation; and (iii) transcripts putatively involved in dormancy*.* Those results suggest that *VvFLC2* and other transcripts with similar expression patterns such as *VvAGL15.1* or *VvSPL14-L* could have a role in bud dormancy regulation in grapevine whereas no evidence for a participation of *VvSVP* genes in this process could be observed.

## Methods

### Plant materials

Grapevine (*Vitis vinifera* L. cultivar Tempranillo) buds were obtained from an experimental vineyard at the Instituto Madrileño de Investigación y Desarrollo Rural, Agrario y Alimentario (IMIDRA, Alcalá de Henares, Madrid). Samples were collected from triplicate blocks in the same vineyard during two consecutive years. Plants and buds developmental stages were classified following the developmental series of Baggiolini (1952) [74] and modified E-L system [75]. Buds were collected at equivalent stem positions from the base and always at the same time of the day. Bud samples were frozen in liquid nitrogen and stored at -80°C before RNA extraction. Samples corresponding to May (MAY), June (JUN), July (JUL), September (SEP), November (NOV), January (JAN), March (MAR) and April (APR) buds were analyzed. Meteorological data were obtained from a station at Finca El Encin (IMIDRA, Alcalá de Henares).

### RNA extraction

Total RNA was extracted from frozen bud samples according to Reid et al., 2006 [76]. RNA purification was performed using the RNeasy Mini Kit (QIAGEN) according to manufacturer's protocols. To remove DNA traces in RNA samples, DNase I digestion was carried out with the RNase-Free DNase Set (QIAGEN). RNA integrity and quantity were assessed by Agilent’s Bioanalyzer 2100. Microarray hybridizations were performed at the Genomics Unit of the National Centre for Biotechnology (CNB-CSIC, Madrid). Raw microarray data from the reported experiments are publicly available at the Plant Gene Expression Database (PlexDB) [77] and labelled as “VV36: Time course of grapevine bud development”.

### Microarray data processing and analysis

Monitoring of bud transcriptional activity was performed in three biological replicates for each time point using Affymetrix Grapegen GeneChip®. Raw Affymetrix CEL files were imported to Robin software suite [78] to perform data normalization using the RMA method. Principal component analysis was performed using Acuity software [79] (Molecular Devices, LLC, CA, US). The score matrix was used to select probe-sets that best fit the first principal component (PC1) and those with PC1 scores greater than 7 or lower than -7 were chosen. Likewise, probe-sets that best fitted PC2 were those with component score greater than 3 or lower than -3.

Differential expression analyses were performed in Multi Experiment Viewer [80] using LIMMA, applying a 0.01 cut-off for *P*-value and log_2_ fold ratio greater than 1 and lower than -1. *P* values were corrected using the Benjamini-Hochberg test.

To identify the biological functions over-represented within selected probe sets functional enrichment analyses were performed using FatiGO [81] (*P*-value< 0.05). Functional categories were based on manual annotation of the custom made GrapeGen GeneChip®, based on 12X v1 grape genome assembly, described in Grimplet et al., 2012 [82].

Key regulators of reproductive development were selected according to their functional annotation [82]. Expression values were extracted from the whole experiment normalized data matrix (averaged from the triplicates per sample and probe set). When more than one probe set matched a single gene transcript, only one was selected. Hierarchical clustering was performed using MultiExperiment Viewer [80] based on Pearson's correlation and using the complete linkage option.

## Competing interests

Authors declare that they have no competing interests.

## Authors’ contributions

JDR and MJC performed the sample collection and RNA extractions. JDR together with JG performed the transcriptomic analysis. MJC drafted the manuscript. MJC and JMZ conceived the study, partook in its design and coordination. All authors worked in developing the final manuscript and read and approved it.

## Supplementary Material

Additional file 1 **Table containing the RMA normalized expression values for all GrapeGen GeneChip^®^ probesets in the analyzed samples together with their correspondences to genes in the 12X V1 version of grapevine reference genome and their annotations.** This file also includes the component scores for each probe-set in the first three principal components. Click here for file

Additional file 2 Table containing the RMA normalized expression values for the probesets selected by PC1 component score (both positive and negative values) in the analyzed samples. Click here for file

Additional file 3 Table containing the RMA normalized expression values for the probesets selected by PC2 component score (both positive and negative values) in the analyzed samples. Click here for file

Additional file 4 **Functional categories significantly enriched in the clusters selected by PC2 component score (both positive and negative values).** Categories enriched in the positive cluster are depicted in green while those enriched in the negative one are in pink. Absolute values of the log_10_ transformed *P*-values were used for the bar diagram representing statistical signification, only categories with *P*-values < 0.05 were shown. Click here for file

Additional file 5 **Expression pattern of the two grapevine *FLC *homologs.** Average expression values for each time-point are shown. Click here for file
