# Supplementary figures and images for: Transcriptome variation along bud development in grapevine (Vitis vinifera L.)
Source: BMC Plant Biol. 2012 Oct 5;12:181. doi: 10.1186/1471-2229-12-181 (PMC3519583; doi:10.1186/1471-2229-12-181)

PC2 significant functions

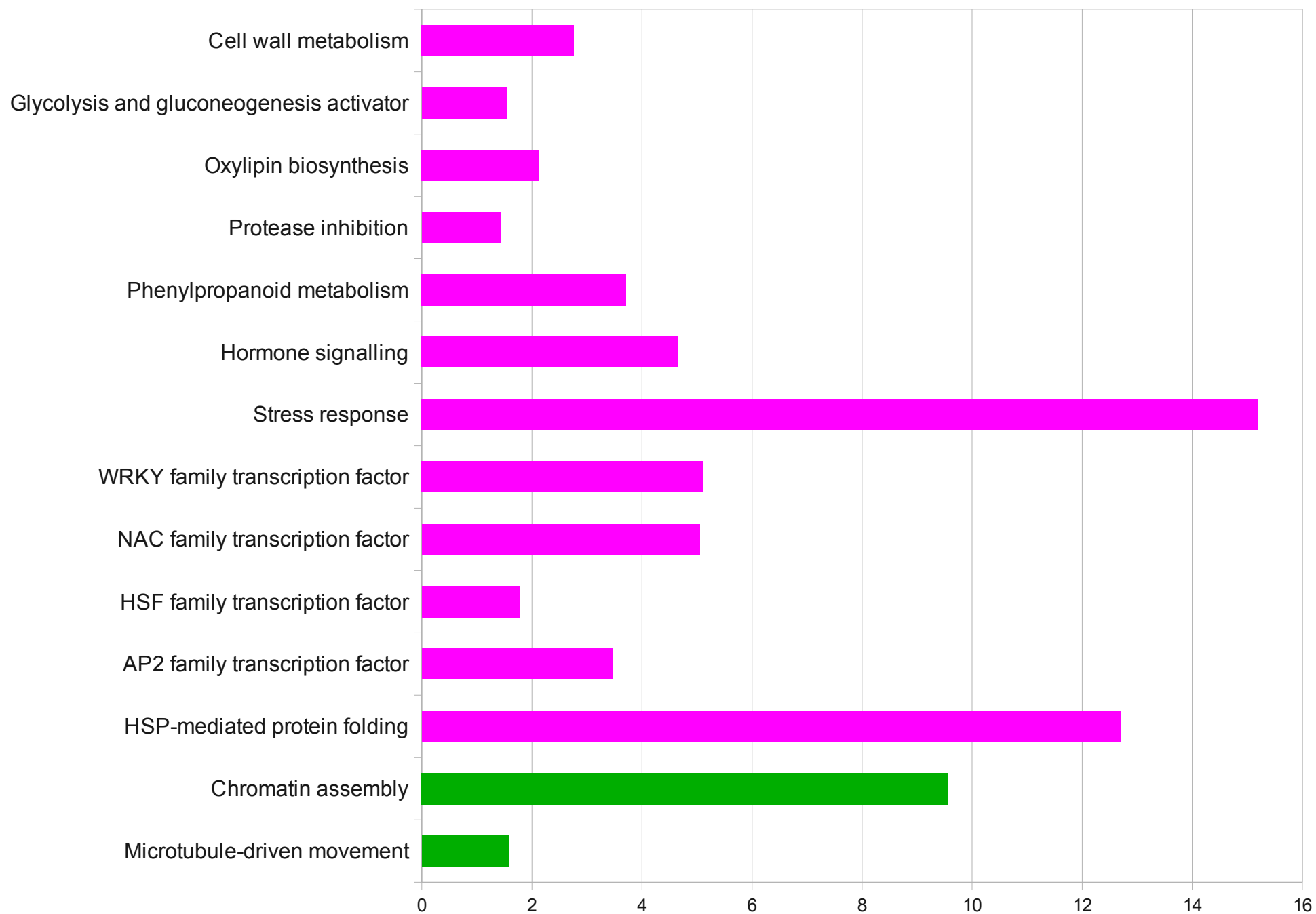

Supplement: Additional file 4 — Functional categories significantly enriched in the clusters selected by PC2 component score (both positive and negative values). Categories enriched in the positive cluster are depicted in green while those enriched in the negative one are in pink. Absolute values of the log10 transformed P-values were used for the bar diagram representing statistical signification, only categories with P-values < 0.05 were shown. [file 1471-2229-12-181-S4.pdf]

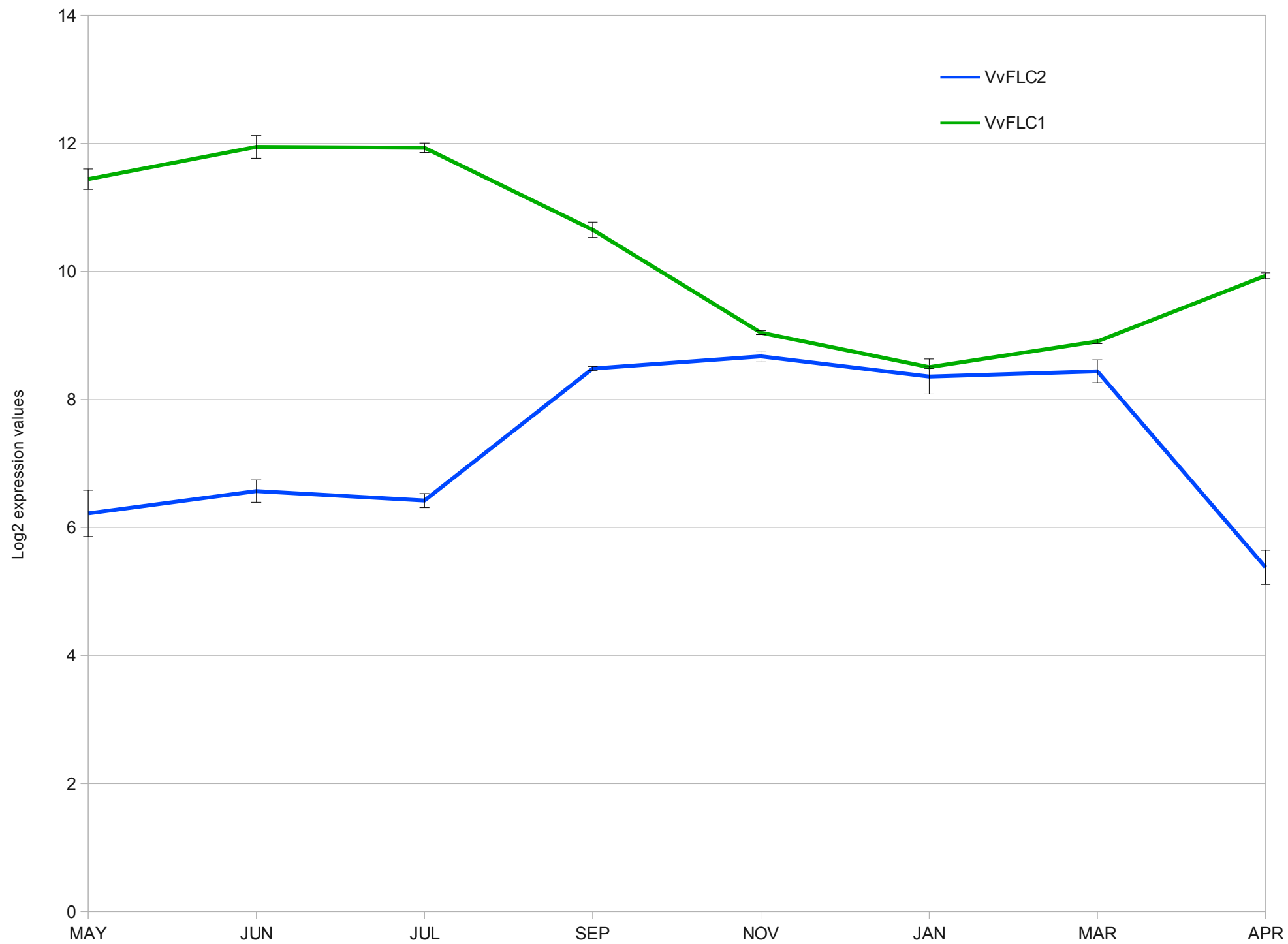

Supplement: Additional file 5 — Expression pattern of the two grapevine FLC homologs. Average expression values for each time-point are shown. [file 1471-2229-12-181-S5.pdf]
